# Noninvasive Optical Monitoring of Cerebral Blood Flow and EEG Spectral Responses after Severe Traumatic Brain Injury: A Case Report

**DOI:** 10.3390/brainsci11081093

**Published:** 2021-08-20

**Authors:** Chien-Sing Poon, Benjamin Rinehart, Dharminder S. Langri, Timothy M. Rambo, Aaron J. Miller, Brandon Foreman, Ulas Sunar

**Affiliations:** 1 Department of Biomedical Engineering, Wright State University, Dayton, OH 45435, USA; poon.4@wright.edu (C.-S.P.); rinehart.32@wright.edu (B.R.); langri.2@wright.edu (D.S.L.); 2 Quantum Opus, LLC, Novi, MI 48375, USA; tim@quantumopus.com (T.M.R.); aaron@quantumopus.com (A.J.M.); 3 Department of Neurology & Rehabilitation Medicine, University of Cincinnati, Cincinnati, OH 45267, USA; foremabo@ucmail.uc.edu

**Keywords:** cerebral blood flow and oxygenation, diffuse correlation spectroscopy, EEG, traumatic brain injury, neurointensive care unit, neuromonitoring

## Abstract

Survivors of severe brain injury may require care in a neurointensive care unit (neuro-ICU), where the brain is vulnerable to secondary brain injury. Thus, there is a need for noninvasive, bedside, continuous cerebral blood flow monitoring approaches in the neuro-ICU. Our goal is to address this need through combined measurements of EEG and functional optical spectroscopy (EEG-Optical) instrumentation and analysis to provide a complementary fusion of data about brain activity and function. We utilized the diffuse correlation spectroscopy method for assessing cerebral blood flow at the neuro-ICU in a patient with traumatic brain injury. The present case demonstrates the feasibility of continuous recording of noninvasive cerebral blood flow transients that correlated well with the gold-standard invasive measurements and with the frequency content changes in the EEG data.

## 1. Introduction

Traumatic brain injury (TBI) is a significant public health burden, contributing to 30% of all injury-related deaths in the U.S. and resulting in long-term disability for more than 3 million Americans at an annual cost of $80 billion [1]. There is a significant interest in developing effective management strategies to improve clinical outcomes. Survivors of severe TBI often require critical care, during which the brain is vulnerable to secondary injuries, including anoxia, ischemia, and edema, that result, in part, from uncoupling cerebral blood flow (CBF) from metabolic demand. A major goal of neurocritical care is to monitor the brain to detect and minimize these secondary brain injuries. There is a need for real-time, noninvasive, multimodal measurements for intervention guidance.

Several clinical technologies for imaging are available, including computed tomography (CT) and magnetic resonance imaging (MRI). While these are noninvasive, imaging provides only single snapshots in time, and they are not suited for continuous, long-term monitoring in the bedside neuro-ICU settings [2]. In contrast, continuous monitoring in most ICUs is limited to continuous assessments of cardiopulmonary function. Invasive probes to measure intracranial pressure (ICP) are recommended for select patients according to the Brain Trauma Foundation Guidelines [1,3]. We recently described a standardized method for multimodality monitoring (MMM) which includes not only ICP but a thermal diffusion flowmetry (TDF) probe (Hemedex, Inc; Cambridge, MA, USA) to measure CBF measurements from brain tissue directly [4]. However, these methods require drilling a burr hole through the skull and placement of probes directly into the cortex, conferring the risk of hemorrhage while sampling only one area of the frontal lobe. Aside from sampling only a small brain volume, TDF also suffers from data loss as a result of automatic recalibration every 30 min. Laser Doppler flowmetry (LDF) measures CBF from a very small volume of tissue, and LDF devices have not been FDA-approved for clinical use in humans [5]. Transcranial Doppler ultrasound (TCD) measures the velocity of flow within large cerebral arteries but cannot directly measure CBF. It is limited by skull thickness, and is challenging to use for longitudinal monitoring due to the need for stable ultrasonic probe orientation [6].

Continuous EEG (cEEG) is capable of detecting seizures, periodic discharges, and spreading depolarizations leading to secondary brain ischemia and may contain relevant prognostic information in patients after moderate or severe TBI [7,8,9,10]. Currently, cEEG monitoring is recommended by the international neurocritical care community for use in patients following TBI. It has been recognized that characteristic changes occur during EEG in response to brain ischemia in correlation with CBF and oxygen metabolism [10,11]. When CBF becomes compromised, the metabolic and electrical activity of cortical neurons is impacted, leading to alternations in the frequency content of the EEG [10]. However, the use of EEG for ischemia monitoring is not widespread despite commercially available quantitative software. Thus, there exists an unfilled niche for continuous, bedside monitoring of cerebral blood flow within the vulnerable cortex in the neuro-ICU setting.

Diffuse optical techniques permit continuous noninvasive measurements of cerebral hemodynamics in microvasculature [6,12]. Near-infrared spectroscopy (NIRS) measures changes in cerebral oxygenation but cannot provide direct CBF continuously [6,12]. Furthermore, oxygen saturation alone may not be sensitive enough to detect secondary brain injury hours after the insult as oxygen consumption and delivery reach equilibrium after acute transients [6,12,13]. There has been significant work on cerebral oxygenation monitoring with NIRS [14,15]. Robertson et al. showed a ~90% sensitivity in detecting secondary hematomas [16]. Kampfl et al. showed that patients with an ICP of >25 mmHg exhibited significantly reduced NIRS parameters than those with an ICP <25 mmHg [17]. However, there are limitations in NIRS monitoring [1,2]. Bush et al. showed that the NIRS approach was limited in detecting cerebral hypoxia in acute brain injury patients when compared to Clark electrode measurements [13]. Leal-Noval et al. found NIRS was reliably sensitive in detecting relatively severe cerebral hypoxia (PbrO 2 < 12 mmHg) but showed a weak correlation between invasive brain tissue oxygen pressure (PbtO2) measurements with NIRS-derived tissue oxygenation [18]. Overall, NIRS studies indicated that there was no strong evidence of significant correlation with the invasive monitoring modality [19]. Almost all used commercial NIRS systems provided information related to changes in oxygenation rather than absolute values, and these changes did not correlate well with the clinical outcome [20]. These systems work in continuous wave (CW)-mode and cannot effectively separate scalp signal from brain signal. A more recently developed diffuse optical technique, diffuse correlation spectroscopy (DCS), probes speckle fluctuations related to blood flow [6,12,21,22,23,24]. It is ideal for high-risk populations, as it can be used as a continuous, noninvasive, portable bedside monitor of CBF [6,13,25,26,27,28,29].

There is a much higher contrast between cerebral blood flow vs. scalp blood flow [29]. Thus, we expect blood flow measurements may provide additional benefits for TBI monitoring [13,29]. In fact, DCS was shown to have a higher sensitivity to the brain compared to NIRS [30]. DCS has been used to provide noninvasive, continuous measurements of CBF in animals and humans [24,31,32,33], and recently, its use in neuro-ICU settings [6,13,25,34] has demonstrated that DCS measurements agree with clinically-established modalities [21,24,33]. Furthermore, we recently demonstrated the potential of using DCS to elucidate resting-state functional connectivity (RSFC) [35,36]. Here we report continuous cerebral tissue blood flow measurements in a TBI patient by using optical diffuse correlation spectroscopy. Clinically-standard electroencephalogram (EEG) auxiliary recordings provided a complementary fusion of data about brain activity and function.

## 2. Materials and Methods

### 2.1. Study Design and Patient Details

This is a case report from a patient with severe TBI admitted to the Neuroscience Intensive Care Unit (NSICU) at the University of Cincinnati, an American College of Surgeons (ACS) level I trauma center. Invasive multimodality neuromonitoring devices were placed per our clinical standard and time-locked with cEEG and systemic physiologic data as previously described. All care was provided according to national (ACS) guidelines. Written informed consent was provided by a legally authorized representative prior to study procedures as approved by the Institutional Review Board at the University of Cincinnati.

**Figure 1:**
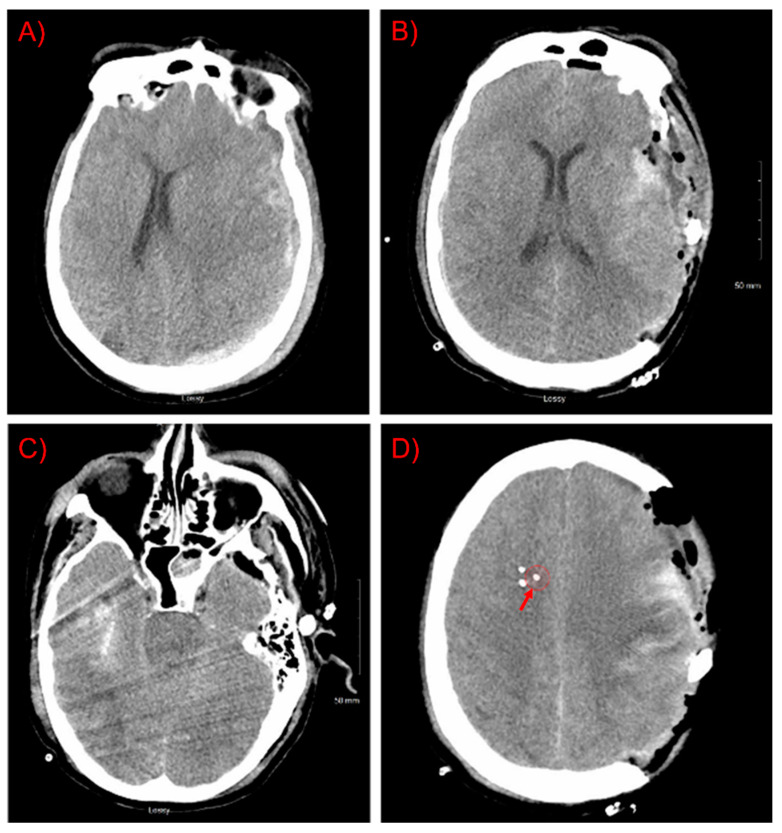

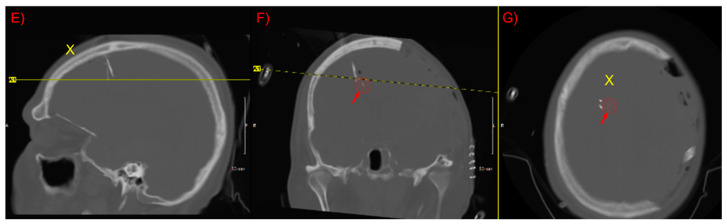
Computed Tomography (CT) head imaging. (**A**) Pre-operative imaging at presentation demonstrated left subdural hematoma with midline shift. (**B**–**D**) Postoperative imaging after decompressive hemicraniectomy and evacuation of the acute hemorrhage demonstrating peri-hematoma contusion but the resolution of the midline shift. An area of right temporal contusion is also seen with relative compression of the cisterns surrounding the brainstem. Intracranial monitoring devices were placed in the contralateral hemisphere approximately 2.5 cm under the inner table of the skull within the subcortical white matter. The location of the Bowman Perfusion probe is highlighted by a red circle. (**E**–**G**) Sagittal, coronal, and axial sections of the postoperative CT imaging windowed to highlight dense structures, including the intracranial monitoring devices. The Bowman Perfusion probe is highlighted with a red circle on coronal and axial sections. The approximate location of the DCS probe is marked with a yellow “X”.

A 43-year-old man with no significant past medical history was admitted after being struck by a motor vehicle. Upon the arrival of Emergency Medical Services, he was noted to have extensor posturing and was intubated in the field. In the Emergency Department, his Glasgow Coma Scale (GCS) score was 3T, and he had bilaterally nonreactive pupils. Noncontrast head CT demonstrated an 8 mm left subdural hematoma with 1.2 cm left-to-right midline shift (Figure 1). Hypertonic saline was given, resulting in the return of bilateral pupillary reactivity and improvement in the clinical exam to 6T. He was taken emergently to the operating room, where he underwent left decompressive hemicraniectomy and clot evacuation. In addition, he was found to have several grade-1 blunt cerebrovascular injuries, cervical spinal hyperextension injury without cord signal change, and multiple orthopedic fractures. On post-trauma day 1 and postoperative day 0, he underwent placement of multimodality intracranial monitoring.

### 2.2. Optical Technique

Optical data were acquired using a custom-built DCS system, as published previously [35,36]. In summary, the custom-made DCS instrument employed a continuous-wave laser source (785 nm CrystaLaser; Reno, NV, USA) with a coherence length longer than 10 m, eight NIR-optimized superconducting Nanowire single-photon counting detectors (QuantumOpus, LLC; Novi, MI, USA), and an 8-channel auto-correlator board (Correlator.com, Bridgewater, NJ, USA). A multi-mode fiber (1000 μm core diameter) was used to guide the 785 nm laser light to the scalp, and a few-mode fiber (8.2 μm core diameter) coupled quantum nanowire single-photon counting modules collected the light. Photodetector outputs were fed into a correlator board that computed light intensity temporal autocorrelation functions that were recorded by a computer using a custom LabVIEW software. We used four DCS detectors at 2.7 cm, three detectors at 2.5 cm to average the signal, and one detector at the shortest separation of 8 mm to obtain the scalp signal. The custom optical probe was based on a foam pad that incorporated prisms, enabling 90-degree light delivery and detection for optimal probe-tissue contact. DCS data were acquired at 1 Hz. A 15 s moving average filter was applied to the signal to increase the signal-to-noise ratio, and the signal was normalized to the first point to obtain a relative measure of blood flow changes.

For the quantification of the CBF using DCS technique, we assumed an optical absorption parameter of 0.15 cm^−1^ and a scattering parameter of 10 cm^−1^. The normalized diffuse electric field temporal autocorrelation function (g_1_ (r,τ)) was extracted from measured normalized intensity temporal autocorrelation function (g_2_ (r,τ)) and then it was fitted to an analytical solution of the diffusion equation to estimate the blood flow index (BFi) parameter.

All clinical monitoring data were exported into a common format (MATLAB) for analysis using CNS Envision (Version 1.00.00) with the CNS Data Conversion plugin (Moberg Solutions, Inc.; Ambler, PA, USA). From EEG recordings, we chose the electrode channel most closely overlying the region studied by the optical probe (F4). We incorporated EEG data from the nearby electrocorticography (ECoG) electrode, choosing the single-channel recording of the highest amplitude and best quality. To get the density spectral array (DSA), we used Welch’s method to generate power spectra with 8 segments and 50% overlap from 1–20 Hz. Each segment was windowed with a Hamming window and over 4 s epochs for EEG and ECoG channels. The power of the theta frequency band (4–8 Hz) was obtained by applying a 1-min moving average filter. The alpha–delta ratio (ADR) [37] was calculated by taking the ratio of the power of the alpha frequency band to the delta frequency band with a 2-min moving average filter.

## 3. Results

Clinical multimodality monitoring initially demonstrated decompressed physiology with resolving cerebral edema. Regional cerebral blood flow (rCBF) from the TDF probe was highly correlated with mean arterial blood pressure (MAP), suggesting impaired regulation, although improvements were seen in the TDF rCBF from post-trauma day 1 to 2. Cerebral micro dialysis demonstrated mitochondrial dysfunction with the development of relatively low interstitial brain glucose concentrations between days 2 and 3. Brain tissue oxygen monitoring did not respond to an increased fractional inspiration of oxygen (FiO2), and therefore PbtO2 values were deemed not accurate measurements of dissolved oxygen tension (PtO2) within the monitored region of tissue. A confirmatory head CT demonstrated adequate probe placement without peri-catheter hematoma (Figure 1).

**Figure 2:**
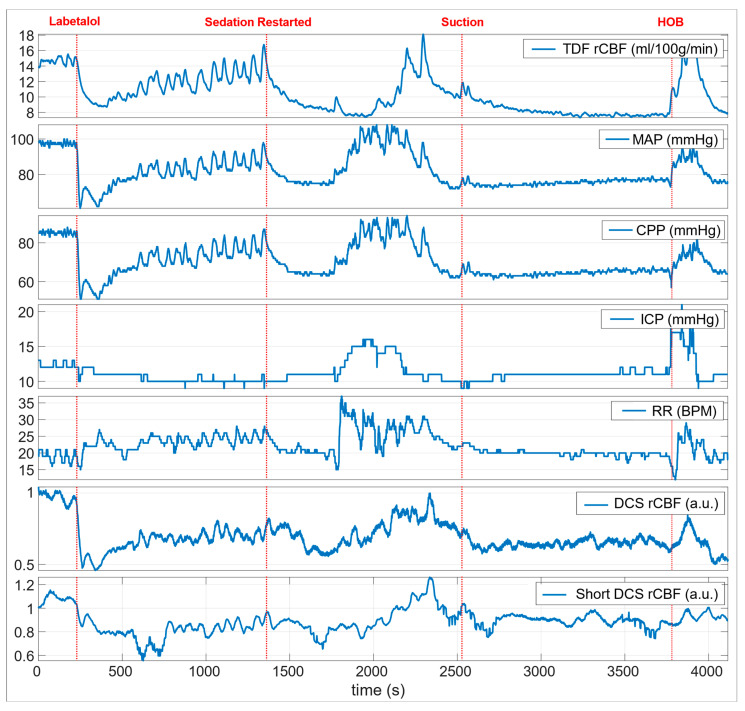
Clinical multimodality monitoring of the TBI patient on post-trauma day 2. The red dashed line indicates clinically relevant events annotated during the care of the patient. The normalized regional cerebral blood flow (DCS rCBF) from the noninvasive optical probe at 2.7 cm is also shown here, along with the short (8 mm) source–detector separation probe (Short DCS rCBF) for measuring scalp flow.

On post-trauma day 2, intravenous administration of labetalol 10 mg resulted in an abrupt decrease in mean arterial blood pressure (MAP; 98 mmHg to 61 mmHg); cerebral perfusion pressure (CPP; 86 mmHg to 51 mmHg); ICP (13 mmHg to 10 mmHg); and heart rate (HR; 105 to 91 bpm) (Figure 2).

**Figure 3:**
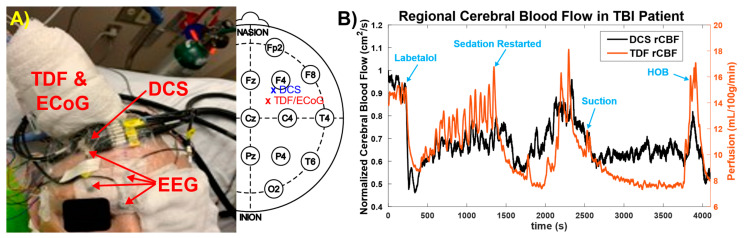
The feasibility of noninvasive optical blood flow measurements after severe traumatic brain injury (sTBI). (**A**) Multimodal invasive probe (including TDF and ECoG), EEG, and noninvasive optical probe placement. The approximate placement of the invasive and noninvasive probes is also shown on an EEG10/20 map; (**B**) Measured blood flow with invasive, thermal-diffusion flowmetry (TDF rCBF) and noninvasive optical imaging (DCS rCBF). Sedation medication led to a substantial blood flow decrease, while HOB manipulation led to a significant blood flow increase, which could be measured by both techniques.

The noninvasive optical probe was placed close to EEG channel F4 to monitor the CBF (Figure 3A). During the optical spectroscopy recording, the optical (DCS rCBF) measurements indicated a similar trend as compared to the TDF rCBF measured with the invasive TDF probe. After administration of labetalol, there was a sharp decrease in both TDF rCBF (42.1%, ~15.1 mL/100 g/min t ~8.74 mL/100 g/min) and DCS rCBF (49.5%, ~0.942 to ~0.476) (Figure 3B). Sedative medications, propofol at 40 mcg/kg/min and fentanyl 200 mcg/h, were paused for a period of approximately 20 min as blood pressure recovered, resulting in vasomotor oscillations in TDF rCBF, MAP, and HR signals as observed in Figure 2. Subsequently, the sedation was restarted where a decrease in TDF rCBF (~42.9%, ~14.3 mL/100 g/min to ~8.16 mL/100 g/min) with a time-locked decrease in DCS rCBF (~28.3%, ~0.770 to ~0.552) was observed (Figure 3B). In the middle of the recording period, an airway suctioning procedure was performed, resulting in a brief increase in TDF rCBF (20.4%, ~9.84 mL/100 g/min to ~11.85 mL/100 g/min) and DCS rCBF (9.29%, ~0.671 to ~0.733) (Figure 3B). Toward the end of the recording period, the head of the bed (HOB) was lowered for patient repositioning, resulting in an increase in MAP and ICP and a doubling of the TDF rCBF (96.8%, ~7.81 mL/100 g/min to ~15.4 mL/100 g/min) (Figure 2). The DCS rCBF simultaneously increased (41.8%, ~0.577 to ~0.818).

**Figure 4:**
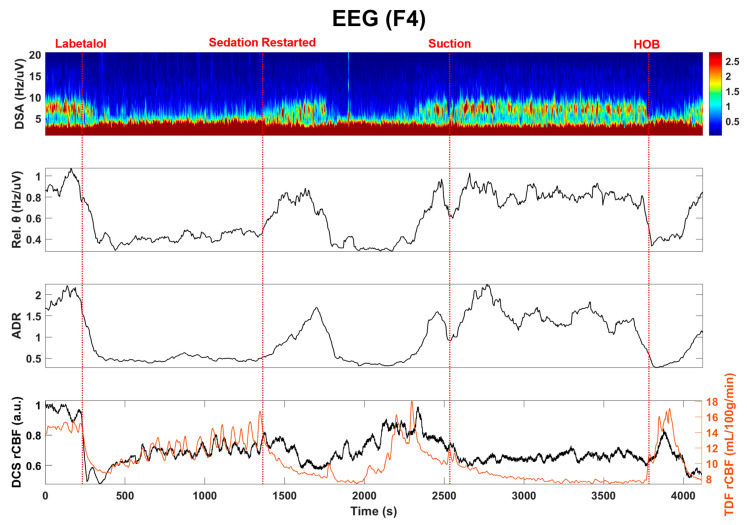
EEG (F4) recording correlates with TDF rCBF. Density spectral array (DSA), theta frequency band and the alpha to delta ratio (ADR) of EEG recordings along with TDF rCBF are shown here. After labetalol administration, the TDF rCBF decreased along with the high-frequency content within the EEG signal, as has been described during ischemia. In contrast, there was increasing high-frequency activity after sedation was restarted despite a decrease in TDF rCBF as might be expected with sedation, such as propofol, which lowers the metabolic demand of the tissue and is associated with diffuse higher frequency activity on EEG. This pattern was interrupted by suctioning and manipulation of the head of the bed, with an inverse relationship between TDF rCBF and higher frequency EEG activity, as might be expected during arousal.

**Figure 5:**
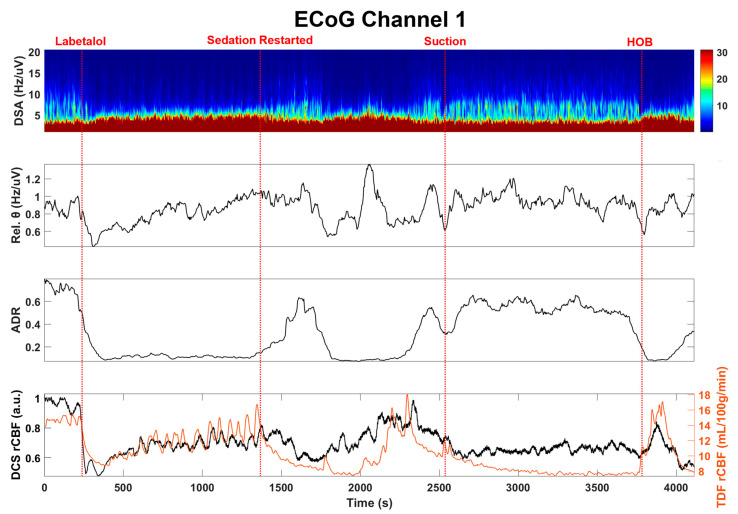
Electrocorticography (ECoG) recording correlates with BF. Density spectral array (DSA), theta frequency band, and the alpha to delta ratio (ADR) of ECoG recordings along with the TDF rCBF is shown here. The relationship between regional blood flow and regional cortical postsynaptic potentials was similar to those observed on EEG.

Next, we examined the EEG and the invasive electrocorticography (ECoG) responses to changes in TDF rCBF, as summarized in Figure 4 and Figure 5. Quantitative EEG signal analysis indicated a reduction in higher frequencies and a decrease in the alpha to delta ratio (ADR, %) and density spectral array (DSA, Hz) [38], which aligned well with both clinical CBF and DCS-derived CBFi. These findings suggest compromised cerebral perfusion pressure (CPP) (Figure 5).

The patient subsequently required open reduction and fixation of the right femur and external fixation of both knees and anterior cervical decompression and fixation of the cervical spine. His hospital course was complicated by persistent respiratory failure with refractory hypoxia thought to be related in part to multi-segment rib fractures and pneumonia. He required tracheostomy and percutaneous gastrostomy prior to discharge to long-term acute care.

## 4. Discussion

We demonstrated here that optical blood flow measurements can provide continuous, noninvasive monitoring of quantitative blood flow that can be coupled with EEG and other multimodality neuromonitoring parameters to provide important insights at the bedside. After sTBI, neurocritical care is aimed at limiting secondary injuries by closely monitoring the brain. Existing methods exhibit limit temporal or spatial sampling, limiting their utility. Optical imaging technologies have been promising, but its commercially available devices do not measure blood flow directly, leading to challenges in interpretation. We demonstrated that our method clearly correlated with invasive measurements of rCBF and further that our measurements informed the interpretation of EEG signals to diagnose a period of relative ischemia and the impact of arousal and sedation.

Invasive ICP monitoring (Figure 5) remains a cornerstone of neurocritical care. However, intracranial monitoring incurs a small but important risk for complications related to placement, and devices are further limited by sampling from only a single region within the brain. Multiple modalities may be important to better understand the state of the monitored tissue relative to isolated measurements of ICP. As such, we previously published our clinical platform for providing multimodality monitoring as part of standard care, including measurements of rCBF using a thermal diffusion flowmetry device [4]. One of our main goals of this pilot study was to show the feasibility of providing continuous, longitudinal measurements of blood flow. In this study, we measured blood flow for approximately two hours, but our technique can continually measure CBF for 24 h or more. A primary limitation was the need for space on the scalp, which may be taken by numerous other devices, both invasive and noninvasive. However, optical fibers can be arranged close to EEG electrodes, as we showed here, and this can reduce the additional space requirements needed and allow for routine correlation of optical metrics with added spatial sampling in conjunction with EEG recordings.

Continuous EEG (cEEG) monitoring is currently recommended to detect electrographic seizures and may be useful for ischemia monitoring [10] based on characteristic changes that occur on EEG in response to decreases in blood flow oxygen metabolism [10,11]. As normal CBF declines, the EEG loses faster frequencies relative to slower frequencies as a crucial ischemic threshold is reached. Indeed, we observed that declines in the relative DCS rCBF (Figure 2) were closely correlated with quantitative TDF rCBF, and both aligned with this expected EEG frequency response (Figure 3 and Figure 4). Importantly, we also highlighted that EEG frequency changes alone are non-specific—with decreases in faster frequency activity that occurred within the context of increased CBF during states of relative stimulation or arousal. Our noninvasive measurements of blood flow were adequate to allow for crucial contextualization of our observations from EEG and ECoG.

Importantly, we highlighted the validity of our noninvasive optical measurements relative to direct, invasive thermal diffusion-based CBF measurements, which correlated well throughout the course of recording and during multiple clinical events. Optical measurements are performed by placing sensors on the scalp, which is a highly vascular tissue that can induce extracerebral signal contamination. Increasing the source–detector separation to probe deeper within a tissue can occur at the cost of a lower signal-to-noise (SNR) ratio. Superconducting nanowire detectors have much higher quantum efficiency (>90%) at ~800 nm compared to traditional single-photon counting modules (e.g., Excelitas, ~60%), which improved our SNR at longer source–detector separations. It should be stressed that this study demonstrated the clinical translation capability of superconducting nanowire detectors for the first time. Adding multiple fibers (multiple detectors) for each position to average the optical signal may further circumvent this issue but at an additional cost for each additional detector. We found that it is preferable that optical probes are adjusted for each patient individually in order to improve SNR at a minimal cost based on the monitoring required. We focused on obtaining good quality of data for this first patient of our clinical study with the traditional NIR wavelength of ~785 nm. In the future, these detectors can be used at longer wavelengths (e.g., 1064 nm), which is expected to increase the signal ~10-fold, based on the previous work [39,40].

Our study has several limitations. First of all, this was mainly a case report, and the results cannot be generalized. TDF measurements are not always reliable as they are very sensitive to placement and require calibration every ~30 min due to signal drifts, which leads to data loss [6]. Another major limitation in our study was the extracerebral contamination coming from scalp flow due to limited penetration of the photon depths at the source–detector separation of 2.7 cm, and it is likely that we have underestimated the CBF changes [6,25,41]. Here we used a homogeneous diffuse reflectance model, but the regression model, as suggested by [6], and the 2-layer model implemented by Baker et al. may improve the brain sensitivity [25].

## 5. Conclusions

We described here a case study where optical imaging using DCS provided continuous, noninvasive blood flow changes which (a) correlated well with direct, invasive measurements of rCBF, (b) integrated with multimodality monitoring data, including ICP, and (c) linked EEG and ECoG with blood flow to highlight changes associated with ischemia, sedation, and arousal. These results support the clinical feasibility of our approach to noninvasive continuous bedside monitoring in the Neuro-ICU setting. In the future, this multimodal approach may provide neurological and physiological information to guide intervention to eliminate the risk of secondary brain injury.

## Data Availability

Data may be available via direct contact via email request.

## References

[1] Zaninotto A.L.C., Costa B.T., Ferreira I.S., French M., Paiva W.S., Fregni F. (2018). Traumatic brain injury. Neuromethods.

[2] Amyot F., Arciniegas D.B., Brazaitis M.P., Curley K.C., Diaz-Arrastia R., Gandjbakhche A.H., Herscovitch P., Hinds S.R., Manley G.T., Pacifico A. (2015). A Review of the Effectiveness of Neuroimaging Modalities for the Detection of Traumatic Brain Injury. J. Neurotrauma.

[3] Le Roux P., Menon D.K., Citerio G., Vespa P., Bader M.K., Brophy G.M., Diringer M.N., Stocchetti N., Videtta W., Armonda R. (2014). Consensus Summary Statement of the International Multidisciplinary Consensus Conference on Multimodality Monitoring in Neurocritical Care. Neurocrit. Care.

[4] Foreman B., Ngwenya L. (2019). Sustainability of Applied Intracranial Multimodality Neuromonitoring After Severe Brain Injury. World Neurosurg..

[5] Kenney K., Amyot F., Haber M., Pronger A., Bogoslovsky T., Moore C., Diaz-Arrastia R. (2016). Cerebral Vascular Injury in Traumatic Brain Injury. Exp. Neurol..

[6] Selb J., Wu K.-C., Sutin J., Lin P.-Y., Farzam P., Bechek S., Shenoy A., Patel A.B., Boas D.A., Franceschini M.A. (2018). Prolonged monitoring of cerebral blood flow and autoregulation with diffuse correlation spectroscopy in neurocritical care patients. Neurophotonics.

[7] Vespa P.M., Nuwer M.R., Nenov V., Ronne-Engstrom E., Hovda D.A., Bergsneider M., Kelly D.F., Martin N., Becker D.P. (1999). Increased incidence and impact of nonconvulsive and convulsive seizures after traumatic brain injury as detected by continuous electroencephalographic monitoring. J. Neurosurg..

[8] Vespa P.M., Miller C., McArthur D., Eliseo M., Etchepare M., Hirt D., Glenn T., Martin N., Hovda D. (2007). Nonconvulsive electrographic seizures after traumatic brain injury result in a delayed, prolonged increase in intracranial pressure and metabolic crisis. Crit. Care Med..

[9] Rosenthal E.S., Ms S.B., Zafar S.F., Ba K.L.O., Bechek S., Shenoy A.V., Bs E.J.B., Shafi M.M., Gilmore E.J., Foreman B.P. (2018). Continuous electroencephalography predicts delayed cerebral ischemia after subarachnoid hemorrhage: A prospective study of diagnostic accuracy. Ann. Neurol..

[10] Foreman B., Claassen J. (2012). Quantitative EEG for the detection of brain ischemia. Crit. Care.

[11] Foreman B., Albers D., Schmidt J.M., Falo C.M., Velasquez A., Connolly E.S., Claassen J. (2017). Intracortical electrophysiological correlates of blood flow after severe SAH: A multimodality monitoring study. Br. J. Pharmacol..

[12] Boas D.A., Franceschini M.A. (2011). Haemoglobin oxygen saturation as a biomarker: The problem and a solution. Philos. Trans. R. Soc. A Math. Phys. Eng. Sci..

[13] Busch D.R., Balu R., Baker W.B., Guo W., He L., Diop M., Milej D., Kavuri V., Amendolia O., Lawrence K.S. (2018). Detection of Brain Hypoxia Based on Noninvasive Optical Monitoring of Cerebral Blood Flow with Diffuse Correlation Spectroscopy. Neurocrit. Care.

[14] Barud M., Dabrowski W., Siwicka-Gieroba D., Robba C., Bielacz M., Badenes R. (2021). Usefulness of Cerebral Oximetry in TBI by NIRS. J. Clin. Med..

[15] Davies D.J., Su Z., Clancy M.T., Lucas S., Dehghani H., Logan A., Belli A. (2015). Near-Infrared Spectroscopy in the Monitoring of Adult Traumatic Brain Injury: A Review. J. Neurotrauma.

[16] Robertson C.S., Gopinath S., Chance B. (1997). Use of near infrared spectroscopy to identify traumatic intracranial hemotomas. J. Biomed. Opt..

[17] Kampfl A., Pfausler B., Denchev D., Jaring H.P., Schmutzhard E. (1997). Near Infrared Spectroscopy (NIRS) in Patients with Severe Brain Injury and Elevated Intracranial Pressure. Brain Edema X 1997.

[18] Leal-Noval S.R., Cayuela A., Arellano-Orden V., Marín-Caballos A., Padilla V., Ferrándiz-Millón C., Corcia Y., García-Alfaro C., Amaya-Villar R., Murillo-Cabezas F. (2010). Invasive and noninvasive assessment of cerebral oxygenation in patients with severe traumatic brain injury. Intensive Care Med..

[19] Esnault P., Boret H., Montcriol A., Carre E., Prunet B., Bordes J., Simon P., Joubert C., Dagain A., Kaiser E. (2014). Assessment of cerebral oxygenation in neurocritical care patients: Comparison of a new four wavelengths forehead regional saturation in oxygen sensor (EQUANOX^®^) with brain tissue oxygenation. A prospective observational study. Minerva Anestesiol..

[20] Spiotta A.M., Stiefel M.F., Gracias V.H., Garuffe A.M., Kofke W.A., Maloney-Wilensky E., Troxel A., Levine J.M., Le Roux P.D. (2010). Brain tissue oxygen–directed management and outcome in patients with severe traumatic brain injury. J. Neurosurg..

[21] Durduran T., Choe R., Baker W., Yodh A.G. (2010). Diffuse optics for tissue monitoring and tomography. Rep. Prog. Phys..

[22] Boas D.A., Yodh A.G. (1997). Spatially varying dynamical properties of turbid media probed with diffusing temporal light correlation. J. Opt. Soc. Am. A.

[23] Boas D.A., Campbell L.E., Yodh A.G. (1995). Scattering and Imaging with Diffusing Temporal Field Correlations. Phys. Rev. Lett..

[24] Mesquita R.C., Durduran T., Yu G., Buckley E.M., Kim M.N., Zhou C., Choe R., Sunar U., Yodh A.G. (2011). Direct measurement of tissue blood flow and metabolism with diffuse optics. Philos. Trans. R. Soc. A Math. Phys. Eng. Sci..

[25] Baker W.B., Balu R., He L., Kavuri V.C., Busch D.R., Amendolia O., Quattrone F., Frangos S., Maloney-Wilensky E., Abramson K. (2019). Continuous non-invasive optical monitoring of cerebral blood flow and oxidative metabolism after acute brain injury. Br. J. Pharmacol..

[26] Kim M.N., Edlow B.L., Durduran T., Frangos S., Mesquita R., Levine J.M., Greenberg J.H., Yodh A.G., Detre J.A. (2013). Continuous Optical Monitoring of Cerebral Hemodynamics During Head-of-Bed Manipulation in Brain-Injured Adults. Neurocrit. Care.

[27] Favilla C., Mesquita R., Mullen M., Durduran T., Lu X., Kim M.N., Minkoff D.L., Kasner S.E., Greenberg J.H., Yodh A.G. (2014). Optical Bedside Monitoring of Cerebral Blood Flow in Acute Ischemic Stroke Patients During Head-of-Bed Manipulation. Stroke.

[28] Mullen M.T., Parthasarathy A.B., Zandieh A., Baker W.B., Mesquita R.C., Loomis C., Torres J., Guo W., Favilla C.G., Messé S.R. (2019). Cerebral Blood Flow Response During Bolus Normal Saline Infusion After Ischemic Stroke. J. Stroke Cerebrovasc. Dis..

[29] Forti R.M., Favilla C.G., Cochran J.M., Baker W.B., Detre J.A., Kasner S.E., Mullen M.T., Messé S.R., Kofke W.A., Balu R. (2019). Transcranial Optical Monitoring of Cerebral Hemodynamics in Acute Stroke Patients during Mechanical Thrombectomy. J. Stroke Cerebrovasc. Dis..

[30] Selb J.J., Boas D.A., Chan S.-T., Evans K.C., Buckley E.M., Carp S. (2014). Sensitivity of near-infrared spectroscopy and diffuse correlation spectroscopy to brain hemodynamics: Simulations and experimental findings during hypercapnia. Neurophotonics.

[31] Durduran T., Yodh A.G. (2013). Diffuse correlation spectroscopy for non-invasive, micro-vascular cerebral blood flow measurement. NeuroImage.

[32] Yu G., Durduran T., Zhou C., Cheng R., Yodh A.G.G. (2011). Near-Infrared Diffuse Correlation Spectroscopy for Assessment of Tissue Blood Flow. Handbook of Biomedical Optics.

[33] Buckley E.M., Parthasarathy A.B., Grant P.E., Yodh A.G., Franceschini M.A. (2014). Diffuse correlation spectroscopy for measurement of cerebral blood flow: Future prospects. Neurophotonics.

[34] Forti R.M., Katsurayama M., Menko J., Valler L., Quiroga A., Falcão A.L.E., Li L.M., Mesquita R.C. (2020). Real-Time Non-invasive Assessment of Cerebral Hemodynamics with Diffuse Optical Spectroscopies in a Neuro Intensive Care Unit: An Observational Case Study. Front. Med..

[35] Li J., Poon C.S., Kress J., Rohrbach D.J., Sunar U. (2017). Resting-state functional connectivity measured by diffuse correlation spectroscopy. J. Biophotonics.

[36] Poon C., Rinehart B., Li J., Sunar U. (2020). Cerebral Blood Flow-Based Resting State Functional Connectivity of the Human Brain using Optical Diffuse Correlation Spectroscopy. J. Vis. Exp..

[37] Claassen J., Hirsch L., Kreiter K.T., Du E.Y., Connolly E.S., Emerson R.G., Mayer S.A. (2004). Quantitative continuous EEG for detecting delayed cerebral ischemia in patients with poor-grade subarachnoid hemorrhage. Clin. Neurophysiol..

[38] Topjian A.A., Fry M., Jawad A.F., Herman S.T., Nadkarni V.M., Ichord R., Berg R.A., Dlugos D.J., Abend N.S. (2015). Detection of Electrographic Seizures by Critical Care Providers Using Color Density Spectral Array After Cardiac Arrest Is Feasible*. Pediatr. Crit. Care Med..

[39] Carp S.A., Tamborini D., Mazumder D., Wu K.-C., Robinson M.R., Stephens K.A., Shatrovoy O., Lue N., Ozana N., Blackwell M.H. (2020). Diffuse correlation spectroscopy measurements of blood flow using 1064 nm light. J. Biomed. Opt..

[40] Colombo L., Pagliazzi M., Sekar S.K.V., Contini D., Durduran T., Pifferi A. (2020). In vivo time-domain diffuse correlation spectroscopy above the water absorption peak. Opt. Lett..

[41] Gagnon L., Desjardins M., Jehanne-Lacasse J., Bherer L., Lesage F. (2008). Investigation of diffuse correlation spectroscopy in multi-layered media including the human head. Opt. Express.

